# A review of marine microalgae-based lipids production: biosynthesis, technological advancements, and practical applications

**DOI:** 10.3389/fnut.2025.1664674

**Published:** 2025-11-11

**Authors:** Limin Yang, Bomingxin Duan, Qian Lu

**Affiliations:** 1 School of Life Sciences, Jiangsu University, Zhenjiang, China; 2 Zhenjiang Fengzhou Zhiyu Biotechnology Co., Ltd., Zhenjiang, China; 3 School of Grain Science and Technology, Jiangsu University of Science and Technology, Zhenjiang, China

**Keywords:** microalgae, unsaturated fatty acids, cultivation, animal feed, lipid

## Abstract

Marine microalgae have emerged as a sustainable and renewable feedstock for lipid production, offering significant potential to address environmental challenges and feed resource scarcity. This review provides a comprehensive analysis of marine microalgae-based lipid production, integrating insights from biosynthesis, technological advancements, and practical applications. First, we elucidate lipid accumulation process in marine microalgae, focusing on metabolic regulation, environmental stressors, and pharmaceutical functions. Next, this paper critically evaluate cutting-edge innovations in marine microalgae cultivation strategies, such as culture medium alternative, two-stage cultivation model, and microalgal cells immobilization. Last, the review highlights diverse applications of microalgal lipids in feed production for aquatic animals, livestock and poultry. Specific effects of dietary supplementation of microalgal lipid on the growth performance, health status and meat quality of animals are summarized. This review also assesses the technical challenges and practical viability of marine microalgae-based lipid production. Accordingly, some potential solutions which will promote the wide application of microalgal lipid in aquaculture and livestock/poultry farming are proposed. It is expected that this review can help researchers gain a more comprehensive understanding of marine microalgal lipids and encourage them to find out more actionable strategies to maximize the ecological and economic potential of marine microalgal lipids.

## 1 Introduction

As one of the essential raw materials in animal feed, lipids play a key role in aquaculture and livestock/poultry farming (1–3). Dietary intake of unsaturated fatty acids are favorable to the health status of animals (4–6). In the past, the production of lipids as an additive in animal feed relied excessively on reduction fishery, which yields fish oil containing long-chain polyunsaturated fatty acid (PUFA). Besides, traditional agriculture, particularly the cultivation of soybean, peanut, oil palm, and sunflower, could provides vegetable oil (7–10). However, global ocean pollution and soil degradation are constraining the sustainable development of lipid production reliant on reduction fishery and traditional agriculture (11, 12). Additionally, the expansion of oil crop cultivation competes with staple food production, creating potential food security risks and threatening the stability of human society (13, 14). Recently, aforementioned problems are intensifying the search for an alternative lipid resource, which can support the sustainable development of aquaculture and livestock/poultry farming.

Among the emerging bio-based technologies, marine microalgae have garnered significant attention as a sustainable and eco-friendly platform to produce a variety of bio-products, including lipids (15, 16). In the feed or food industry, lipids containing functional components, such as PUFAs, natural antioxidants, and functional pigments, are widely used. These lipids have more beneficial effects on the health of animals or humans and possess higher market value. Therefore, lipids containing functional components have garnered increasing attention in both scientific research and industrial sectors. Marine microalgae, a diverse group of photosynthetic microorganisms naturally living in ocean and coastal regions, exhibit unparalleled advantages over terrestrial crops (e.g., soybean, peanut, sunflower, etc.) for the production of lipids (17). Firstly, marine microalgae have much higher growth rate and shorter cultivation period than terrestrial oil crops. It was reported that the cultivation period of marine microalgae ranged between 10 and 20 days while that of soybean could reach several months (18–20). According to the experimental results in laboratory research, compared to terrestrial oil crops, microalgae demonstrate 10–20 times higher oil yield per unit area, making them a scalable solution for large-scale lipid production. Secondly, cultivation of marine microalgae for lipid production does not intensively compete with traditional agriculture for arable land, posing no threat to the food security of human society. Thirdly, some marine microalgae contain much more PUFA, such as eicosapentaenoic acid (EPA), docosahexaenoic acid (DHA), and α-linolenic acid (ALA), which are of importance to the health status and growth performance of animals (21, 22). For example, dietary intake of PUFA could scavenge the reactive oxygen species in cells and improve the immunity of animals. In recent years, as the functional benefits of marine microalgal lipids become increasingly recognized, feed industry, which traditionally focused on fish oil, have expanded their microalgal lipid product offerings, leading to a burgeoning array of health-promoting microalgal lipid.

In fact, as early as several decades ago, researchers had reported the presence of abundant lipids in marine microalgae. However, in the field of science and technology, the transition from laboratory research to industrial implementation requires a holistic understanding of the technological and socioeconomic factors (23, 24). Key knowledge gaps persist in elucidating the regulatory mechanisms of lipid metabolism under stress conditions, optimizing large-scale cultivation systems, and developing high-value bio-products using microalgal lipids. Hence, it was not until recent technological advancements that microalgal lipid production gradually transitioned from theoretical potential to practical reality, now actively contributing to the development of the bio-based economy (25, 26). In the past several years, researchers conducted extensive studies in the field of microalgal lipid production, with the primary focuses covering lipid synthesis, microalgae cultivation, and practical application of microalgal lipids. Particularly, as carbon neutrality is becoming a necessary condition of the achievement of Paris Agreement temperature goals, carbon bio-sequestration by employing microalgae lipid production has garnered growing attention in recent research (27–29). According to the data, soy oil production in 2015 was 48.8 million ton, which accounted for 29% of global vegetable oil production (30). Hence, it was estimated that 168.3 million ton vegetable oil was produced in 2015. Given the present states of technology and production capability, microalgal lipids remain unable to fully substitute fish oil or vegetable oils as principal lipid sources. Given the advantages of marine microalgae in synthesizing PUFA, they are primarily positioned as a lipid production platform rather than mere substitutes for vegetable oil.

This review provides a comprehensive analysis of the current state of marine microalgae-based lipid production, with a focus on three interconnected themes: (1) the metabolic pathways and environmental triggers governing lipid biosynthesis in marine microalgae, (2) cutting-edge technological innovations for marine microalgae cultivation, and (3) practical applications of marine microalgal lipids as feed additives in the aquaculture and the livestock and poultry farming. It should also be noted that despite the technological advances, commercialization of marine microalgal lipids faces multifaceted challenges, including safety risks of biomass, poor digestibility and palatability, and uncertainty of new models. By summarizing recent research advances and identifying persistent challenges, this work aims to guide future research and industrial efforts toward realizing the full potential of marine microalgae as a sustainable lipid source in the bio-based economy. Compared with publications which mainly focused on either microalgal lipid synthesis, cultivation techniques, or feed production technology, this review provides a comprehensive analysis of the aforementioned aspects from an industry chain perspective. Through integrating unsaturated fatty acids induction, microalgae-based wastewater remediation, and dietary supplementation of microalgal lipids, This review presents to readers the application of microalgae in PUFA production, wastewater treatment, and animal farming across the integrated industrial chain. In the view of the present authors, the main innovations of this review include: (1) discussing emerging technologies for marine microalgae-based lipid production from the perspective of industrial chain development; (2) examining the advantages of microalgae lipid as a carbon capture carrier for wastewater remediation; and (3) providing a comprehensive analysis of the applications of marine microalgae lipid in aquaculture and livestock farming.

## 2 Lipid production in marine microalgae

In the field of fundamental research, scientists primarily focus on three key aspects: the synthesis of lipid and lipid-soluble components, the relationship between lipid synthesis and carbon bio-sequestration, and environmental factors influencing microalgal lipid synthesis.

### 2.1 Synthesis of lipid and lipid-soluble components

#### 2.1.1 Lipid and polyunsaturated fatty acid

As one of the critical cellular constituents, lipids serve fundamental metabolic functions in microalgal metabolism. Accumulation of lipid, which contains more energy than starch and protein, as a major survival strategy favoring the microalgal cells exposed to cold environment has recently been demonstrated (31). Also, lipid degradation is essential for microalgae to remodel membrane lipids and mobilize storage lipids, enabling survival and growth during environmental fluctuations (32).

In fact, although microalgae from both marine environment and freshwater environment contain PUFA, marine microalgae are more widely employed as the producers of lipids. Main reasons for this phenomenon include the vast marine cultivation environment, high PUFA content, and diverse algal species in ocean. Firstly, the rapid growth of global population has led to increasing pressure on terrestrial resources, making freshwater microalgae cultivation on land potentially competitive with traditional agriculture. In contrast, the vast marine environment offers sustainably developed space for microalgae cultivation. This is one of the key reasons for utilizing marine microalgae as a bio-carrier for lipid production. Secondly, marine microalgae have higher PUFA content and lipid productivity. According to the survey of 19 brackish and marine microalgae, average lipid content of dry weight was around 22% and some microalgae even contained up to 40.6% lipid content (33). In marine microalgae, lipid synthesis, starting from carbon fixation via the Calvin cycle or glycolysis, primarily occurs in chloroplasts and endoplasmic reticulum. In addition, compared with freshwater microalgae, marine microalgae contain much higher content of long-chain PUFA, such as ARA (Arachidonic acid), DHA and EPA (34, 35). In nature, enrichment of long-chain PUFA in lipid can be regarded as a self-protection mechanism of marine microalgae in low-temperature environment. The comparatively lower freezing point of PUFA enhances the cold tolerance of marine microalgae by maintaining the membrane fluidity and improving their survival rates in low-temperature environments. Thirdly, marine microalgae encompass a much greater diversity of species than freshwater microalgae, thus offering a broader selection for industrial applications. Considering the advantages of marine microalgae in the production of lipids, in this review paper,.

Mechanism study has revealed that the biosynthesis of PUFA in marine microalgae initiates with the elongation and desaturation of precursor fatty acids, primarily starting from saturated fatty acids, such as palmitic acid (36–38). Two groups of membrane-bound enzymes, elongases and desaturases, are driving elongation and desaturation, respectively (39). Notably, final forms of fatty acids by the end of elongation and desaturation process not only influenced by genetic expression, but also regulated by environmental factors (40). In addition, polyketide synthase (PKS) pathway, which is an oxygen-independent pathway widely documented in Dinophytes or Thraustochytrids, also exist to produce PUFA (41). The elucidation of multiple metabolic routes has bolstered the practical feasibility of metabolic engineering approaches for boosting lipid biosynthesis in microalgae.

In addition to environmental factors and metabolic pathways, advances in research techniques have progressively identified key regulatory genes controlling microalgal lipid synthesis (38, 42). However, given the stringent regulatory constraints on the application of genetic modification techniques, environmental factors are still the major factors that can be adjusted to enhance the PUFA biosynthesis in marine microalgae.

#### 2.1.2 Lipid-soluble components

Lipids derived from some marine microalgae not only have higher concentrations of PUFA, but also contain more anti-oxidants, such as zeaxanthin, carotene, lutein and fucoxanthin (22, 43, 44). Recently, some lipid-soluble components derived from marine microalgae have been scientifically validated as essential for the growth and health of humans and animals.

As one of the typical lipid-soluble components, astaxanthin with superior antioxidant capacity has been intensively studied to reveal its biosynthesis pathway. Glyceraldehyde 3-phosphate is produced in microalgal cells through glycolysis or the Calvin cycle, and then converted to isopentenyl pyrophosphate, which is the precursor of β-carotene (45). Astaxanthin synthesis pathway various in different microalgal species and the intermediates of synthesis process include echinenone, canthaxanthin, adonirubin, zeaxanthin, and adonixanthin. Finally, a portion of the synthesized astaxanthin is combined with fatty acids to form astaxanthin esters, which can be used as food or feed additive in downstream industry (46, 47).

Pharmaceutical functions of some lipid-soluble components in marine microalgae have been intensively studied. For example, it was reported that some antioxidants, such as β-carotene and astaxanthin, dissolved in lipids can effectively eliminate free radicals and reduce oxidative stress within cells. Theoretically, supplementation of these antioxidants in animal feeds could prevent the peroxidation in cells and delay the cellular aging. In addition, a couple of indirect pharmaceutical functions of lipid-soluble components were reported. For example, astaxanthin from marine microalgae can be added in feeds to increase the growth rate of aquatic organisms, thus preventing the use of synthetically produced hormone. Different from the synthetic chemicals, natural astaxanthin at a reasonable addition level in feed has no negative effects on the health status of aquatic animals. Accordingly, dietary supplementation of astaxanthin can enhance the growth of aquatic animals and improve their survival rate, performing an indirect pharmaceutical function.

### 2.2 Carbon bio-sequestration

Lipids, particularly fatty acids with a long carbon skeleton, serve as excellent carriers of carbon element, playing a key role in carbon bio-sequestration. Percentage of carbon element in microalgal compounds can be estimated according to the equation as follows:


$$\begin{array}{l} {\text{P}}_{\text{C}}=\frac{{\text{N}}_{\text{C}}\times 12}{\text{M}}\times 100\% \end{array}$$

where *P*_*C*_ is the percentage of carbon element; *N*_*C*_ refers to the number of carbon atom in certain microalgal compounds; *M* is the relative molecular weight.

As shown in Table 1, percentages of carbon element in lipid compounds are high, falling in a scope of 69.8%−80.5%. Particularly, percentages (75.0%−80.5%) of carbon element in the common microalgal fatty acids are much higher. By contrast, percentages of carbon element in amino acids and polysaccharide are lower. For example, percentages of carbon element in cysteine and starch are only 29.8% and 44.4%, respectively (Table 1). On an equal mass basis, lipid-rich marine microalgae capture and sequester more carbon compared to those rich in protein or polysaccharides. Therefore, lipid-rich marine microalgae can be regarded as a promising carbon sink, capturing both carbon-containing organics in culture medium and atmospheric CO_2_.

**Table 1:** Estimation of the percentage of carbon element in major microalgal compounds.

**Category**	**Name of certain microalgal compound**	**Chemical formula**	**Relative molecular weight**	**Total number of carbon atoms**	**Percentage of carbon element**
Fatty acids	Docosahexaenoic acid	C_22_H_32_O_2_	328	22	80.5%
	Docosapentaenoic acid	C_22_H_34_O_2_	330	22	80.0%
	Eicosapentaenoic acid	C_20_H_30_O_2_	302	20	79.7%
	Arachidonic acid	C_20_H_32_O_2_	304	20	79.0%
	α-Linolenic acid	C_18_H_30_O_2_	278	18	77.7%
	Linoleic acid	C_18_H_32_O_2_	280	18	77.1%
	Oleic acid	C_18_H_34_O_2_	282	18	76.6%
	Palmitic acid	C_16_H_32_O_2_	256	16	75.0%
	Palmitoleic acid	C_16_H_30_O_2_	254	16	75.6%
Other forms of lipids	Sterol lipid	C_17_H_28_O (Sterol)	248	17	82.3%
	Prenol lipid	C_5_H_10_O (Prenol)	86	5	69.8%
Amino acids	Aspartic acid	C_4_H_7_NO_4_	133	4	36.1%
	Serine	C_3_H_7_NO_3_	105	3	34.3%
	Cysteine	C_3_H_7_NO_2_S	121	3	29.8%
	Glycine	C_2_H_5_NO_2_	75	2	32.0%
Polysaccharides	Starch	C_6_H_10_O_5_	162	6	44.4%
	Cellulose	C_6_H_10_O_5_	162	6	44.4%

### 2.3 Environmental factors

Environmental factors which can significantly influence the lipid metabolism in marine microalgae include the nutrient profile of culture medium, microalgae cultivation temperature, and illumination characteristics.

#### 2.3.1 Nutrient profile of culture medium

Since nitrogen and phosphorus assimilation is closely related to protein synthesis and carbon metabolism, adjustment of nutrient concentration in culture medium can change the trophic mode, particularly the synthesis of protein and lipid in marine microalgae. Past studies confirmed that through conducting nitrogen or phosphorus starvation, lipid content in marine microalgae could be improved (18, 48). In addition, the type of organic carbon can influence the lipid content in microalgae. For example, when the C/N ratio in culture medium was set as 15:1, microalgae supplied with acetate as the carbon source contained 18.2% lipid in dry biomass while those supplied with dextrose only contained 8.7% lipid (49).

In recent years, researchers have conducted more in-depth mechanistic studies to elucidate the effect of nutrient-deficiency on lipid composition and relevant metabolism in marine microalgae (50, 51). In the study of marine microalga, *Diacronema lutheri*, grown in nitrogen-deficiency environment, it was observed that the neutral lipid content increased significantly while glycolipid, phospholipid, and betaine lipid content decreased during the 11-day cultivation period (52). Therefore, nutrient stress not only affects total lipid content, but also modify the lipid composition. Additionally, although both nitrogen deficiency and phosphorus deficiency can enhance the lipid synthesis in marine microalgae, it was observed phosphorus starvation caused enlargement of cell size and increased carbon content of *Tisochrysis lutea* while nitrogen starvation had not similar effect on the cell size (50). The enlargement of cell size may be related to perturbation in the progression of cell-cycle (50, 53). In the future, with the elucidation of more lipid metabolism mechanisms, researchers will be able to more effectively improve the lipid quality of marine microalgae through nitrogen and phosphorus deficiencies.

In a real-world application, negative effect of nutrient deficiency on microalgae biomass production, which has no direct relation with lipid content but determines lipid productivity and lipid yield, should not be neglected. It was reported that with the application of nitrogen depletion, biomass productivity of marine microalga, *Dunaliella salina*, dropped dramatically (54). As a consequence, although nitrogen deficiency improved lipid content in *Dunaliella salina*. from ~26% to ~48%, lipid productivity decreased slightly (54). Negative effects of nitrogen deficiency on the growth rates of marine microalgae, *Dunaliella tertiolecta* and *Thalassiosira pseudonana*, were also reported (55).

#### 2.3.2 Cultivation temperature

Lipid content in marine microalgae is negatively correlated with temperature increase. In the study of growing marine microalga, *Chaetoceros* sp., at different temperatures, when the temperature increased from 25 °C to 40 °C, lipid content of *Chaetoceros* sp. Decreased from 20.4% to 8.0% and lipid productivity dropped from 66.7 to 15.9 mg/L/day (56). Negative effects of temperature increase on lipid content were also observed in the culture of many other marine microalgae, such as *Tetraselmis suecica, Nannochloropsis* sp., and *Porosira glacialis* (56, 57). Significantly, low temperature impairs microalgal growth kinetics, leading to detrimental effects on overall lipid productivity and lipid yield. This necessitates a strategic equilibrium between cellular lipid content and total lipid output in the psychrophilic cultivation systems.

Not only lipid content, but also fatty acid composition in microalgal lipid is impacted by the cultivation temperature. In general, the fatty acids of marine microalgae grown at lower temperature exhibit a high degree of unsaturation. When the cultivation temperature increased from 8 °C to 26 °C, percentage of PUFA in the lipid of *Nannochloropsis salina* decreased from 14.50% to 9.48% while percentage of saturated fatty acid (SFA) increased from 33.9% to 46.7% (58). It was observed that with the increase of temperature from 8 °C to 24 °C, percentage of PUFA significantly dropped from 40.1% to 6.4% and percentage of EPA decreased from 34.8% to 3.5%, confirming the positive contribution of low temperature to PUFA synthesis (59). The main reason for this phenomenon is that to maintain their membrane fluidity at lower temperature, marine microalgae incorporate higher levels of PUFA in membrane lipids (60, 61). It should be noted that there are exceptional cases in which the percentage of PUFA demonstrates a positive correlation with temperature elevation. For example, the increase of temperature from 8 °C to 20 °C resulted in the increase of PUFA percentage of *Nannochloropsis oculata* from 17.5% to 26.9% while did not caused significant improvement of SFA percentage (58). Therefore, in the practical application, the relationship between PUFA synthesis and temperature in different marine microalgal species requires case-by-case analysis.

#### 2.3.3 Illumination characteristics

A variety of illumination characteristics, such as light wavelength, light intensity and photoperiod, exert a critical influence on lipid synthesis of marine microalgae. Illumination could determine lipid accumulation through impacting the photosynthesis of microalgae (62). Firstly, specific spectral irradiation can significantly augment lipid synthesis and accumulation in marine microalgae. Compared to red light and white light, blue light has been demonstrated as a superior light source in stimulating marine microalgae to produce elevated levels of lipids (63). Moreover, it was observed that green light of light-emitting diode (LED) has obvious advantages over blue light and red light for lipid accumulation in four marine microalgae, *Phaeodactylum tricornutum, Isochrysis galbana, Nannochloropsis salina*, and *Nannochloropsis oceanica* (64). Green light of LED also significantly improved the percentage of UFA in the aforementioned microalgal strains from 14.3%−21.6% to 23.4%−38.1% (64). Secondly, lipid content in marine microalgae can be improved by optimizing light intensity. During the cultivation of marine diatom, *Amphiprora* sp., increase of light intensity from 6 to 24 μmol/m^2^/s resulted in the improvement of lipid content from 16.5% to 52.5% while excessive light intensity (>24 μmol/m^2^/s) reduced lipid content (65). Similarly, lipid contents in *Isochrysis galbana, Nannochloropsis oculata*, and *Dunaliella salina* reached peak values when the light intensity increased to 150 μmol/m^2^/s (66). Thirdly, photoperiod, which can change the photosynthesis of microalgal cells, is another important factor influencing the lipid synthesis of marine microalgae. According to the experimental results, the maximum total lipid content (31.3%) in *Nannochloropsis* sp. was obtained when microalgae were exposed to 18 h light and 6 h dark while 24:0 light/dark regime and 12:12 light/dark regime resulted in lower lipid content (67). It was reported that when photoperiod were 12:12 light/dark, 24:0 light/dark, and 0:24 light/dark, lipid contents of *Pavlova lutheri* were 35%, 30%, and 15%, respectively (68). Hence, neither constant illumination nor complete darkness has been demonstrated to be conducive to lipid biosynthesis and accumulation in marine microalgae. It is also noteworthy that percentage of PUFA in the lipid of marine microalgae could be improved by the optimized photoperiod (69).

In culture medium supplied with organic carbon as the major carbon source, illumination has no obvious effect on lipid synthesis in microalgae. As reported by Silaban et al., lipid contents in microalgae supplied with acetate and dextrose had not significant difference (49). The main reason for this phenomenon is that microalgae are prone to utilize organic carbon for cellular metabolism and lipid synthesis if there are sufficient organic carbon in culture medium. Under this situation, the role of photosynthesis-driven inorganic carbon uptake in the lipid synthesis process will be diminished. Accordingly, supply of illumination did not significantly improve lipid content in microalgae (49).

## 3 Technological advancements in microalgae-based lipid production

To bridge the gap between lab-scale innovation and industrial deployment, concerted efforts have been dedicated to three key technological dimensions: cost mitigation strategies for large-scale production, innovation of cultivation models for lipid productivity improvement, and streamlining cultivation protocols to improve process scalability.

### 3.1 Wastewater-based microalgae cultivation

#### 3.1.1 Selection of wastewater for marine microalgae cultivation

The cost of artificial culture medium accounts for 30%−45% of the total microalgae cultivation cost. By contrast, some wastewater enriched with nitrogen, phosphorus, metal ions, and organic carbon, which are essential nutrients to microalgae growth, poses a serious threat to the environment. Under this situation, the use of wastewater to replace artificial culture medium for microalgae cultivation not only reduces the cost of microalgal biomass, but also yields profound environmental sustainability impacts.

To our knowledge, wastewater used to cultivate lipid-rich marine microalgae as animal feed additives must meet specific standards. Otherwise, serious environmental contamination and health risk may be caused (70, 71). Firstly, wastewater should contain no toxic compounds, such as heavy metals, toxic chemicals, and excessive ammonia. On one hand, toxic compounds may limit the growth of marine microalgae or even result in the failure of microalgae-based lipid production. For example, when the initial free ammonia concentration in culture medium reached 13.3 mM, the growth of marine microalga, *Chlorella vulgaris*, was inhibited (72). On the other hand, heavy metals are absorbed by microalgae, threatening the health of consumers through the bio-accumulation in food chain. It was reported that due to the functional groups (e.g., hydroxyl, carboxyl, methylene groups, etc.) on the surface of microalgal cells, heavy metal ions with positive changes can be adsorbed, resulting in the contamination of microalgal biomass (73). Therefore, to rule out the negative effects of toxic compounds on the utilization of microalgal biomass as animal feed additives, the safety of wastewater should be controlled strictly. Secondly, wastewater with balanced nutrient profile can be employed for marine microalgae cultivation. Since the low ratio of N/C is favorable to the accumulation of lipid in algal cells, wastewater with higher total organic carbon (TOC) concentration should be used for marine microalgae-based lipid production (74). It should also be noted that digestibility of organic carbon in wastewater should be taken into consideration. Thirdly, wastewater should be obtained at a low cost for marine microalgae cultivation, thus enhancing the market competitiveness of microalgal lipid products. For example, microalgae cultivation plant can be co-established with food processing factory, which produces a huge amount of organics-rich wastewater. Compared with *ex-situ* treatment, *in-situ* treatment of wastewater by microalgae cultivation can significantly reduce the wastewater transportation cost.

#### 3.1.2 Wastewater pretreatment for microalgae cultivation

Up to now, food processing wastewater and agricultural wastewater with high safety level have been widely regarded as a potential artificial medium alternative for marine microalgae cultivation (75–77). To put the concept of growing marine microalgae in wastewater into practice, a couple of technical problems, such as low salinity, growth-limiting factors, nutrient deficiency, should be addressed.

Different from freshwater microalgae, marine microalgae naturally live in seawater environment with high salinity. However, most wastewater from food industry and agriculture does not contain sufficient salt for the survival of marine microalgae. For example, in the study of Wang et al., salinity of seawater and artificial culture medium reached 2.7% while that of wastewater was 0% (19). As a result, although wastewater contained more nutrients, particularly total nitrogen (TN), total phosphorus (TP), and chemical oxygen demand (COD), than artificial culture medium, biomass yield of marine diatom *Phaeodactylum tricornutum* grown in wastewater was only around 0.25 g/L (19). Accordingly, microalgae-based nutrient removal in wastewater was negatively impacted. In a real-world application, sea salt or seawater could be mixed with wastewater to create a comfortable environment for marine microalgae (19, 34).

In addition to the mixture with sea salt or seawater, dilution is widely applied to pretreat wastewater before the inoculation of marine microalgae. Reyimu et al. discovered that specific growth rates of *Tetraselmis suecica* in original wastewater and 25% wastewater were 0.1488 and 0.4778 d^−1^, respectively (78). Positive effect of wastewater dilution was also observed in the cultivation of *Nannochloropsis oculata*, of which the specific growth rate was highest in the 75% diluted wastewater (78). The main reason for this phenomenon is that dilution can reduce the concentrations of some growth-limiting factors, such as high turbidity, ammonia toxicity, high osmotic pressure, and so on. For example, high ammonia concentration (>0.725 mM) in wastewater has inhibitory effects on microalgae of Diatomophyceae (79). Compared with freshwater microalgae, marine diatoms are more sensitive to high concentration of ammonia in culture medium (79). Therefore, appropriate dilution, which could reduce the concentration of ammonia, is necessary to be applied for wastewater pretreatment.

Balancing nutrient profile is an effective way to alleviate the deficiency of nutrient in some wastewater, enhancing microalgae growth and wastewater remediation. As mentioned above, low ratio of N/C in culture medium or wastewater is favorable to the lipid accumulation in microalgal cells. Ge et al. increased the adding amount of leftover dough hydrolysates from 0 to 50.0 mg/L, resulting in a significant improvement of lipid yield from 54.6 to 2,436.0 mg/L and DHA yield from 3.8 to 341.3 mg/L (35). In this case, starch-rich leftover dough hydrolysates with very low N/C ratio modified nutrient profile of the culture medium, providing marine microalgae with more organic carbon for lipid synthesis.

#### 3.1.3 Microalgae-based lipid production and nutrient removal

In terms of microalgae growth and lipid production, some wastewater can be much more promising nutrient source than artificial culture medium. Malibari et al. compared the growth of five marine microalgal species in artificial medium and shrimp farm wastewater, discovering that this wastewater was more favorable to the lipid accumulation in algal cells (80). In this study, total lipid contents of *Chlorella* sp., *Dunaliella* sp., *Navicula* sp., and *Tetraselmis* sp. grown in artificial medium (f/2 medium) were only 6.3, 35.3, 19.3, and 762.3 pg/cell, respectively. However, when these marine microalgae were cultivated in shrimp farm wastewater, their total lipid contents reached 968.0, 238.3, 613.0, and 4667.7 pg/cell. Compared with f/2 medium, wastewater improved the total lipid of *Chlorella* sp., *Dunaliella* sp., *Navicula* sp., and *Tetraselmis* sp. by 152.7, 5.8, 30.8, and 5.1 times, respectively.

As shown in Table 2, marine microalgae removed over 50% nitrogen and phosphorus in food processing wastewater and agricultural wastewater, demonstrating the practical feasibility of integrating microalgae cultivation with wastewater remediation. For example, *Dunaliella salina* even removed 72.5% COD, 84.8% nitrogen, and 80.5% phosphorus in saline food industry wastewater during 15-day period (77). In the view of the present authors, this is attributed to the high growth rate of marine microalgae and the strong biodegradability of food processing wastewater and agricultural wastewater.

**Table 2:** Cultivation of marine microalgae in wastewater for nutrient removal and biomass production.

**Wastewater**	**Microalgae**	**Nutrient removal**			**Biomass production**	**Reference**
		**TOC**	**TN**	**TP**		
Saline food industry wastewater	*Dunaliella salina*	72.5% COD *^*a*^*	84.8% NO^3+^	80.5% PO^4+^	~6 × 10^5^ cells/mL	(77)
Dairy wastewater from milk producing factory	*Tetraselmis suecica*	40.2%	44.9%	42.2% ${\text{PO}}_{4}^{3-}$	0.6 g/L	(34)
Marine aquaculture wastewater	*Isochrysis galbana*	/	>60% DIN *^*b*^*	100% DIP *^*c*^*	/	(76)
Marine aquaculture wastewater	*Chlorella* sp.	/	>90% DIN	100% DIP	/	(76)
Shrimp farm wastewater	*Navicula* sp.	/	/	/	77.6 mg/L	(80)
Shrimp farm wastewater	*Tetraselmis* sp.	/	/	/	81.3 mg/L	(80)
Shrimp farm wastewater	*Navicula* sp.	/	/	/	86.3 mg/L	(80)
Fishery wastewater	*Thalassiosira pseudonana* and *Isochrysis galbana*	/	85.3%	92.8%	1.4 g/L	(141)
Milkfish culture wastewater	*Isochrysis galbana*	/	97.9% NH_3_-N; 94.3% NO_2_-N *^*d*^*	59.4%	132.4–174.7 mg/L/day	(75)
Milkfish culture wastewater	*Nannochlorum* sp.	/	84.1% NH_3_-N *^*e*^*; 93.4% NO_2_-N	71.65%	94.8–711.0 mg/L/day	(75)
Milkfish culture wastewater	*Tetraselmis tetrahele*	/	99.2% NH_3_-N; 97.5% NO_2_-N	51.55%	136.0–187.5 mg/L/day	(75)
Molasses	*Dunaliella salina*	/	/	/	~2.1 g/L	(142)
Starch-rich food processing waste of Chinese steamed bread	*Isochrysis galbana*	/	/	/	3.9 g/L	(143)
Synthetic wastewater	*Dunaliella tertiolecta*	/	/	54.4%	9.6 g/L	(144)
Synthetic wastewater	*Nannochloropsis gaditana*	/	/	33.3%	12.9 g/L	(144)
Municipal wastewater	*Phaeodactylum tricornutum*	89.9% COD	82.2%	96.0%	1.0 g/L	(19)

^*a*^ COD, Chemical oxygen demand; ^*b*^ DIN, Dissolved inorganic nitrogen; ^*c*^ DIP, Dissolved inorganic phosphorus; ^*d*^ NO_2_-N, Nitrate-nitrogen; ^*e*^ NH_3_-N, Ammonia-nitrogen.

Considering the aforementioned economic and ecological benefits of wastewater-based marine microalgae growth, some nutrient-rich wastewater without toxic risks can be exploited as a cost-saving and effective alternative of artificial culture medium for marine microalgae cultivation and lipid production.

### 3.2 Advanced cultivation models

#### 3.2.1 Two-stage cultivation of microalgae

Under conventional approaches, the synthesis of PUFA-rich lipids in marine microalgae is induced by applying stress conditions, such as low temperature or nitrogen deficiency, while the synthesis of carotenoid in marine microalgae is normally enhanced through high-salinity stress or intensive illumination (Table 3). However, the aforementioned environmental factors enhance the accumulation of lipid or lipid-soluble components in marine microalgae at the expense of biomass production (45). For example, low content of dissolved oxygen (DO) enhanced DHA synthesis in marine microalga, *Schizochytrium limacinum*. However, the cultures of *Schizochytrium limacinum* controlled at 50% DO saturation produced a cell density of 181 million cells/mL while the cultures with 10% DO produced only 98.4 million cells/mL (81).

**Table 3:** Two-stage cultivation model for lipid production by marine microalgae.

**Microalgae**	**Culture medium**	**Targeted components**	**Inducing condition**	**Cultivation period**	**Yield of lipid or lipid-soluble components**	**Reference**
*Dunaliella salina*	Food waste hydrolysate	Lipid and carotenoids	High light treatment and high concentration of organic carbon	16 days (Stage 1: 12 days; Stage 2: 4 days)	Lipid content: 0.8 g/L; Carotenoids content: 0.5 g/L	(18)
*Tetraselmis* sp.	Simulated dairy wastewater	Lipid	73.5 mg/L NaNO_3_, 14.7 mg/L NaH_2_PO_4_, 3.5% salinity and 6.14 pH	/	Lipid content: 51.7%	(145)
*Phaeodactylum tricornutum*	Mixed municipal wastewater	Lipid	Supplementation of CO_2_ (5% CO_2_)	12 days (Stage 1: 8 days; Stage 2: 4 days)	Lipid productivity: 54.8 mg/L/day	(19)
*Dunaliella* sp.	Digested poultry litter wastewater	β-carotene	Salinity (180 psu)	24 days (Stage 1: 12 days; Stage 2: 12 days)	β-carotene yield: 7.3 mg/L	(87)
*Isochrysis galbana*	f/2 medium	Lipid	Low-salt stress (10 psu)	14 days (Stage 1: 12 days; Stage 2: 4 days)	Lipid content: 29.0%	(84)
*Dunaliella salina*	f/2 medium	Lipid	Low-salt stress (10 psu)	11 days (Stage 1: 10 days; Stage 2: 1 day)	Lipid content: 40.0%	(84)
*Dunaliella salina*	Modified Johnson's medium	Carotenoids	High light intensity (110 μmol/m^2^/s)	20 days	Carotenoids productivity: 3 mg/L/day (24 pg/cell)	(146)
*Dunaliella tertiolecta*	f/2 medium	Lipid	Supplementation of selenite (10–40 mg/L)	15 days (Stage 1: 9 days; Stage 2: 6 day)	5.73-fold increase of lipid content	(82)
*Dunaliella tertiolecta*	/	Lipid	Salt stress (2.5 M) and supplementation of sodium azide (50 μM)	/	Single-cell lipid content: 70.5% higher than that of control	(147)
*Isochrysis zhangjiangensis*	Seawater with f/2 medium	Lipid	Supplement interval of nitrogen-repletion (24 h)	10 days (Stage 1: 4 days; Stage 2: 6 day)	Lipid content: 40.9%; Lipid productivity: 140.9 mg/L/day	(86)
*Skeletonema costatum*	Seawater with f/2 medium	Lipid and PUFA	High CO_2_ level (5% CO_2_)	5.25 days (Stage 1: 5 days; Stage 2: 6 h)	PUFA *^*a*^* content: 11.8% of total fatty acid; Lipid productivity: 18.4 mg/L	(83)
*Schizochytrium limacinum*	790 By+ medium	DHA	Low dissolved oxygen content (10%)	/	DHA *^*b*^* content: 6.6 g/L	(81)
*Skeletonema costatum*	Seawater with f/2 medium	Lipid	Nitrogen deficiency (6.8 μmol/L) and silicon deficiency (0.36 μmol/L)	5.5 days (Stage 1: 5 days; Stage 2: 12 h)	Lipid content: 26.0 mg/L	(85)
*Nitzschia laevis*	LDM medium	Fucoxanthin	Blue-white mixed light	/	Fucoxanthin productivity: 16.5 mg/L/day	(148)
*Haematococcus pluvialis*	BG-11 medium with sodium chloride	Lipid and astaxanthin	High content of sodium chloride (2 g/L)	21 days (Stage 1: 8 days; Stage 2: 13 day)	Lipid content: 51.7%; Astaxanthin content: 25.9 mg/g	(149)
*Chlorella vulgaris*	BG-11 medium	Lipid and carotenoids	High contents of sodium chloride (10 g/L) and magnesium chloride (5 g/L)	35 days (Stage 1: 15 days; Stage 2: 20 day)	Lipid productivity: 15.6 mg/L/day; Total carotenoids content: 4.4 μg/mL	(150)

^*a*^ PUFA, Polyunsaturated fatty acid; ^*b*^ DHA, Docosahexaenoic acid.

In fact, the high accumulation of PUFA and other antioxidative components in marine microalgae under stress conditions can be considered a self-protection mechanism. For example, at low temperature, to prevent the solidification of membrane lipids, microalgae cells tend to synthesize PUFA to lower the solidification point of lipids, thereby enhancing their survival rate. Meanwhile, due to the negative effects of low temperature, the proliferation metabolism of microalgae cells will be constrained, leading to a decrease in biomass productivity. Due to the self-protection mechanism under environmental stress, marine microalgae achieved high accumulation of PUFA and other antioxidative components at the expense of biomass production. To attenuate the negative effects of the environmental stress on marine microalgae, a two-stage cultivation model has been developed. In the first stage, marine microalgae are cultivated in a comfortable environment without any stress conditions. During this period, although microalgae do not rapidly accumulate lipids or lipid-soluble components, their growth rate can be maintained at a relatively high level, resulting high biomass productivity and yield. In the second stage, stress conditions are applied to induce the synthesis of lipid or lipid-soluble components in marine microalgae. Microalgae growth is limited while the contents of lipid or lipid-soluble components could be significantly improved. Hence, the second stage can be regarded as a process of adding values of the microalgal lipids.

Recent studies primarily focused on exploring the technical advantages of two-stage cultivation over single-stage cultivation, optimizing novel inducing conditions for marine microalgae, and investigating the practical application of two-stage cultivation in real-world production (Table 3). Firstly, previous study compared the applications of single-stage cultivation and two-stage cultivation for lipid production, discovering that with the supplementation of selenite at a density of 20 mg/L, lipid content of *Dunaliella tertiolecta* in single-stage cultivation increased 2.39-fold while that in tow-stage cultivation increased 5.73-fold (82). The advantage of two-stage cultivation over single-stage cultivation was also reported in the study of growing *Skeletonema costatum* for PUFA production (83). In the study of Ra et al. (84), the transfer of *Dunaliella salina* from Stage 1 to Stage 2 resulted in a rapid increase of lipid content (40.0%). It should be noted that dry cell weights of marine microalgae in Stage 2 were not negatively impacted by the inducing conditions (84). These discoveries confirmed that two-stage cultivation model can realize the high accumulation of lipids, PUFA, and lipid-soluble components in marine microalgae without compromising the microalgae biomass production. Secondly, a couple of innovative inducing conditions have been developed to cultivate marine microalgae for lipid and lipid-soluble components production. For example, silicon deficiency was adopted to induce the lipid synthesis in *Skeletonema costatum*, resulting in an increase of lipid productivity by 113.7% compared with the control group without stress condition (85). Besides, supplement interval of nitrogen-repletion was adjusted to enhance lipid synthesis in *Isochrysis zhangjiangensis* (86). Thirdly, two-stage cultivation model has progressively transitioned from laboratory research to industrial-scale application. This novel model was implemented in the integrated marine microalgae cultivation and wastewater (food waste hydrolysate, digested poultry litter wastewater, mixed municipal wastewater, etc.) remediation (18, 19, 87). It was discovered that compared with the lipid productivity of marine microalgae in Stage 1, that of marine microalgae in Stage 2 was improved dramatically, reaching 54.8 mg/L/day (19). These achievements confirmed the practical feasibility of employing marine microalgae to produce lipid by two-stage cultivation model in the industry.

It is noteworthy that some inducing conditions are unfavorable to the cellular metabolisms of marine microalgae. It was discovered that selenite concentrations ranging from 2.5 to 20.0 mg/L caused the lipid peroxidation, reflected by the thiobarbituric acid reactive substances (TBARS) content, in *Dunaliella tertiolecta* during both single-stage and two-stage cultivation models, emphasizing the selenite-induced oxidative stress accompanied by the increased lipid accumulation in microalgal cells (82). Besides, excessive low temperature, which enhances the synthesis of PUFA in marine microalgae, may lead to the formation of intracellular ice crystals, thereby causing a certain degree of damage to the physical structure of marine microalgal cells (88). Therefore, in practical applications of two-stage cultivation model, efforts should focus on minimizing the negative impacts of inducing conditions on cellular metabolism and cellular structure of marine microalgae.

#### 3.2.2 Immobilization of marine microalgae

It was estimated that microalgae harvesting process could account for 20%−30% of total cost (89). Immobilization technology has been regarded as an effective approach to simplifying microalgae harvesting process and reducing biomass production cost. Generally, innovative microalgae immobilization technologies intensively studied in recent years can be classified into three major categories, namely alginate-based immobilization, rotating algal biofilm, and filamentous fungal pelletization.

Firstly, alginate is a kind of polymer matrix, which can form beads ranging from hundreds of micrometers to millimeters in size and be cross-linked with microalgal cells for immobilization (90). In practical applications, large-sized alginate bead with unicellular marine microalgae are harvested efficiently by simple filtration. Up to now, alginate has been successfully employed for the immobilization of a variety of marine microalgae, such as *Nannochloropsis* sp., *Phaeodactylum tricornutum, Chaetoceros gracilis, Thalassiosira weissflogii, Cylindrotheca* sp., and so on (90–92). It is noteworthy that alginate-based immobilization could maintain the high growth rate of marine microalgae and improve the nutritional value of microalgal lipid. It was reported that in alginate bead with the optimized compositions, specific growth rates of *Phaeodactylum tricornutum* reached around 1.2 day^−1^ (90). It was also discovered the enhancement in DHA (0.7%), EPA (7.7%), and PUFA (11.1%) due to immobilization of marine microalgae in alginate beads (91). Promisingly, this immobilization technology has been successfully adopted for wastewater remediation, provides opportunities for coupling the immobilized production of microalgal lipids with nutrient recovery from wastewater (92). In the downstream application, sodium alginate is regarded as a feed additive (maximum concentration: 30,000 mg/kg feed) safe for consumers and environments (93).

Secondly, rotating algal biofilm refers to a new microalgae cultivation designed to make microalgae attached on the substratum, which is normally made of cotton materials (cotton duct, cotton rag, cotton denim, cotton corduroy, etc.) and lignocellulosic materials (pine sawdust, rice husk, oak sawdust, sugarcane bagasse, etc.) (94, 95). Based on the types of intercellular forces, formation of microalgal biofilm on attachment substrate is consisted of two main steps, namely initial adherence by physical adsorption and biofilm thickening by microbial extracellular polymeric substances (96, 97). In the first step, attachment of microalgae occurs when the repulsive electrostatic interaction is overcome by the attractive van der Waals and acid-base interactions (97). In the second step, microalgae and other microorganisms (bacteria, fungi, etc.) attached on the surface can secret extracellular polymeric substances (EPS), which act as “glue” to increase the intercellular adhesion and accelerate the process of biofilm thickening. With the formation of mature microalgal biofilm, a complex ecosystem with various synergistic relationships, such as pH balancing and intercellular exchange of O_2_ and CO_2_, gradually takes shape. By the end of microalgae cultivation on biofilm, biomass can be directly harvested using scrappers, while the residual microalgae on the substratum surface remain viable for continued growth, enabling continuous microalgae production. According to the survey, biomass productivity of microalgae on biofilm could reach 1.3–10.9 g/m^2^/day (97).

Thirdly, filamentous fungi-based pelletization is another potential method to realize the microalgae immobilization. Due to the fungal filamentous structure, which provides numerous binding site for microalgal cells, microalgae can be attached on the surface of fungal pellets. According to the immobilization process, this model can be classified into two categories, fungal pellet-assisted microalgae immobilization (FPA) and fungal spore-assisted microalgae immobilization (FSA) (98). In the model of FPA, filamentous fungi pelletize prior to their application in microalgae adsorption and immobilization. In the model of FSA, fungal spores are inoculated with microalgae together and then form spherical pellets with microalgae embedded within. Adsorption efficiency of microalgae in both FPA and PSA models could be higher than 90% (98, 99). In practical applications, by the end of microalgae cultivation, microalgal-fungal pellets ranging from millimeters to centimeters in size are harvested by filtration or sedimentation. In addition to the microalgae attachment on fungal pellets, the selection of filamentous fungi for microalgae immobilization has also become a key focus of current research. Recently, some edible filamentous fungi, such as *Rhizopus oligosporus, Aspergillus oryzae, Neurospora intermedia*, and *Monascus purpureus*, with nutritional components have been selected to form pellets. With the application of edible fungi to immobilize microalgae, the harvested biomass can be directly use as raw material for animal feed production. Particularly, in this technical route, some edible filamentous fungi not only provide attachment carrier, but also contain protein, lipid, and various natural pigments and antioxidants. For instance, *Aspergillus oryzae*, which has been successfully utilized for microalgae immobilization, contain kojic acid, γ-aminobutyric acid, chitin-glucan complexes, and other components favorable to the health of animals (99, 100). Therefore, employment of filamentous fungi for microalgae immobilization is considered as a novel technology with the potential to upgrade microalgae cultivation models.

### 3.3 Selection standards of marine microalgae species

There are countless species of marine microalgae, but only a limited number are suitable for industrial production of lipids. In the view of the present authors, to put the aforementioned technologies for microalgal lipids production into practice, microalgal species, which are crucial for lipid productivity, cost, and market value, should be selected properly. Factors that should be taken into consideration mainly include the content of target components, robustness to harsh environment, and presence of toxic components. Firstly, marine microalgal species with high contents of PUFA, astaxanthin, β-carotene or other lipid-soluble components should be screened and then applied for industrialization. Up to now, marine microalgal species, which have been widely employed for lipid production in the industry, include *Dunaliella salina, Nannochloropsis* sp., *Schizochytrium* sp., and so on. It should also be noted that in the foreseeable future, with the advancement of technologies, more and more marine microalgal species will be available for lipid production. For example, in recent years, synthetic biology technologies have been widely adopted to regulate the lipid synthetic pathways in microalgal cells, thus improving the productivity or lipid-soluble components (101). Secondly, the selected marine microalgal species should be robust to the harsh environment. Different from artificial culture medium, wastewater may have some growth-liming factors, such as low pH value, high turbidity, and unbalanced nutrient profile. Only by selecting marine microalgal species that are tolerant to harsh conditions can they be cultivated in wastewater to achieve the recovery of nutrients from the wastewater. Thirdly, marine microalgae used as animal feed ingredient should no contain any toxic components, which not only threaten the health of animals, but also accumulate in the food chain. For instance, intake of microcystins, which are commonly found in some marine algae, may result in the gastrointestinal symptoms of mammals (102). In this case, if marine microalgae containing toxins are selected as the producers of lipids for animal farming, the growth of animals may severely challenged.

## 4 Practical applications of microalgal lipids

Practical application of microalgal lipids in downstream industries is is a critical factor in determining both the economic viability and industrial scalability of microalgae-based oil production. Various solutions, such as biofuel production, edible oil, and animal feed additives, were proposed for the industrial application of plant-based and microalgae-based oil (103, 104). In the practice, fluctuations of global crude oil prices limits the development of microalgae-based biofuel and low consumer acceptance of marine microalgal lipids as the alternative of traditional edible oils (e.g., soybean oil, peanut oil, and palm oil) results in the low consumption of microalgal lipids in the food sector. Therefore, exploitation of microalgal lipid as animal feed additive is playing an increasingly important role in the marine microalgae industry.

### 4.1 Use of microalgae lipid for aquaculture

#### 4.1.1 Inclusion of marine microalgae in aquafeed

In recent years, substitution of fish oil or vegetable oil by microalgal lipid for aquafeed production is being intensively studied in both academics and industry. This phenomenon is attributed to the recession of reduction fishery, supply chain stability, high nutritional value of microalgal lipid, and land use efficiency. Firstly, fish oil is primarily derived from wild-caught fish, which has been heavily exploited and overfished in some marine regions. Particularly, overfishing will disrupt marine food webs and result in unbalanced ecological system. Secondly, fisheries harvesting is seasonal while microalgae production is less affected by seasons. Accordingly, supply chain of microalgal lipid for aquafeed use is more stable than that of fish oil. Thirdly, compared with vegetable oil, microalgal lipid contains more long-chain PUFA, which may enhance the immune response and improve the health status of aquatic animals. Fourthly, microalgae cultivation can be conducted in non-arable land for lipid production. From the perspective of agricultural sustainability, microalgal oil holds greater advantages over vegetable oil.

Marine microalgae can be added into aquafeed in the form of extracted lipid, biomass powder, or commercial bio-product to substitute fish oil or vegetable oil (Table 4). For example, lipid was extracted from *Nannochloropsis* sp. and then added in aquafeed to replace pollack liver oil (4). However, in the practical application, extraction process not only leads to lipid loss, but also increases the overall cost of aquafeed production. In most cases, therefore, marine microalgae are added into aquafeed in the form of biomass powder. Normally, omniferous and herbivorous fish could efficiently digest microalgae-based diet and absorb nutrients from microalgae. However, it should also be noted that cellulose, pectin, and other compounds with low digestibility in microalgae biomass may negatively impact the digestive metabolism of carnivorous fish and increase feed conversion ratio (FCR). Recently, with the commercial value of microalgae being increasingly explored, lipid-rich microalgae bio-products are being developed and utilized as an oil source in aquafeed. Three commercial bio-products, ALL G RICH, DHA GOLD, and MO060, made in USA, Switzerland, and France, respectively, were employed to completely replace sardine oil in the diet of gilthead sea bream (105).

**Table 4:** Application of marine microalgal lipid as aquafeed ingredient for aquaculture.

**Microalgae**	**Application purpose**	**Addition amount**	**Aquatic animal**	**Specific effects**	**Reference**
*Pavlova viridis* & *Nannochloropsis* sp.	Substitution of fish oil as an *n*-3 PUFA *^*a*^* source	*Pavlova viridis*: 96.3 and 192.5 g/kg; *Nannochloropsis* sp.: 42.2 and 84.4 g/kg	Juvenile European sea bass (*Dicentrarchus labrax*)	(1) Substitution of total fish oil by192.5 g/kg *Pavlova viridis* resulted in the highest SGR *^*b*^* of sea bass and lowest FCR *^*c*^*; (2) Aquafeed supplemented with 192.5 g/kg *Pavlova viridis* had the highest EPA *^*d*^* content; (3) Histological analyses of liver and intestine samples did not reveal the negative effect caused by the aquafeed with marine microalgae.	(108)
*Schizochytrium* sp.	Substitution of fish oil	4, 8, 12.5, and 16.1 g/100 g	Juvenile Nile tilapia (*Oreochromis niloticus*)	(1) *Schizochytrium* sp. had higher percentage of PUFA than fish oil, improving PUFA content in fish diet; (2) Complete substitution of fish oil by marine microalgae increased weight gain of fish and reduced FCR; (3) Feed intake of fish was reduced with the increase of addition amount of marine microalgae in fish diet; (4) Compared with fish fed basal diet, fish fed diets with marine microalgae had higher PUFA content in filets.	(151)
*Aurantiochytrium* sp.	*n*-3 PUFA source	1, 3, 5, 7, 9, and 11% in diet	*Trachinotus ovatus*	(1) Addition of marine microalgae increased PUFA content, particularly DHA *^*e*^* content, in diet; (2) Substitution of fish meal by marine microalgae resulted in an increase of crude fat content in diet; (3) WGR and SGR of fish were improved by the increase of marine microalgae content in diet; (4) Compared with basal diet, diet with marine microalgae resulted in higher PUFA content in the liver of fish; (5) Intake of the diet with marine microalgae enhanced immunity of fish by increasing the numbers of white blood cells, lymphocyte, granulocyte, and red blood cells in blood.	(21)
*Pavlova* sp.	Substitution of fish oil and fish meal	100 and 200 g/kg	Atlantic salmon (*Salmo salar*)	(1) Addition of *Pavlova* sp. increased PUFA content in fish diet; (2) Diet supplemented with marine microalgae negatively impacted weight gain of fish while did not resulted in low SGR and FCR; (3) Dietary intake of *Pavlova* sp. reduced sterol content in the muscle of fish and also reduced the contents of PUFA, DHA, and EPA.	(109)
/	Substitution of fish oil	0.7% and 3.5% in diet	Gilthead seabream (*Sparus aurata*)	(1) Substitution of fish oil by microalgal oil and vegetable oil did not negatively impact survival, SGR and FCR of fish; (2) Substitution of fish oil by microalgal oil resulted in low contents of DHA and EPA in diet, but increased the ratio of DHA to EPA; (3) Supplementation of 3.5% microalgal oil in diet improved PUFA content in fish; (4) Addition of microalgal oil in diet significantly reduced malondialdehyde content; (5) Substitution of fish oil by microalgal oil reduced the contents of dioxin and dioxin-like polychlorinated biphenyls in feed and filets; (6) Sensory properties of fish filets were not significantly changed by microalgal oil in diet.	(152)
*Schizochytrium* sp. & *Nannochloropsis* sp.	Substitution of fish oil and soybean oil	45.6 g/kg *Schizochytrium* sp. & 68.0 g/kg *Nannochloropsis* sp.; 91.1 g/kg *Schizochytrium* sp. & 136.0 g/kg *Nannochloropsis* sp.	Juvenile olive flounder (*Paralichthys olivaceus*)	(1) Diet with marine microalgae contained much higher PUFA content than that with soybean oil; (2) Compared with soybean oil, marine microalgae increased the ratio of *n*-3/*n*-6 in diet; (3) Substitution of fish oil and soybean oil in diet by marine microalgae did not negatively impact SGR, FCR, and DFI *^*f*^*; (4) Substitution of fish oil by marine microalgae did not significantly change the contents of dorsal muscle lipid and liver lipid of fish; (5) Substitution of soybean oil by marine microalgae in diet increased PUFA content and ratio of *n*-3/*n*-6 in the muscle tissue of fish.	(110)
*Nannochloropsis* sp.	Replacement of pollack liver oil by microalgal powder	10, 40, and 70 g/kg	Post-larval kuruma shrimp (*Marsupenaeus japonicus*)	(1) Substitution of fish liver oil by 70 g/kg microalgal powder did not significantly reduce PUFA content in diet; (2) Inclusion of 40 g/kg microalgal powder in diet improved weight gain and SGR; (3) Contents of total lipid and crude protein in whole-body composition of shrimp were improved by the inclusion of microalgal powder in diet; (4) Microalgal powder significantly improved PUFA content in neutral lipid fraction of whole body of postlarvae shrimp.	(4)
*Nannochloropsis* sp.	Replacement of pollack liver oil by microalgal lipid	2, 8.2, and 14.3 g/kg	Post-larval kuruma shrimp (*Marsupenaeus japonicus*)	(1) Substitution of fish liver oil by 14.3 g/kg microalgal lipid resulted in higher PUFA content in diet; (2) Inclusion of microalgal lipid in diet resulted in lower weight gain, feed intake, and SGR, but increased survival rate of shrimp; (3) Inclusion of 8.2, and 14.3 g/kg microalgal lipid in diet increased total lipid content in whole-body composition of shrimp; (4) Microalgal lipid significantly improved PUFA content in neutral lipid fraction of whole body of postlarvae shrimp.	(4)
/	Replacement of fish oil by PUFA-rich microalgae products	6.0% in diet	Gilthead sea bream (*Sparus aurata*)	(1) Microdiets with three microalgae products had much higher contents of *n*-3 PUFA and *n*-6 PUFA and lower content of MUFA *^*g*^* than diet with fish oil; (2) Two microalgae products improved total length and body weight of fish larvae; (3) Dietary intake of feeds with three microalgae products increased the contents of *n*-3 PUFA and *n*-6 PUFA in fish larvae; (4) Dietary intake of three microalgae products affected the expression of *fads2*, which regulates long chain PUFA biosynthesis.	(105)
*Schizochytrium* sp.	Replacement of fish oil and fish meal	28.8, 58.7, and 88.5 g/kg	Pacific white shrimp (*Litopenaeus vannamei*)	(1) Addition of marine microalgae improved long chain PUFA content while reduced MUFA content in diet; (2) The contents of *n*-3 PUFA and *n*-6 PUFA in the tail issue of shrimp was improved; (3) Marine microalgae enhanced enzymatic antioxidant capacity in the hepatopancreas of Pacific white shrimp.	(15)
*Nannochloropsis* sp.	Replacement of pollack liver oil by microalgal powder	12, 42, and 70 g/kg	Larval kuruma shrimp (*Marsupenaeus japonicus*)	(1) Microalgal powder improved survival rate of larval shrimp to 75.6-82.0%; (2) Compared with the control group, shrimp fed the diet with 70 g/kg microalgal powder had higher final body weight.	(4)
*Nannochloropsis* sp.	Replacement of pollack liver oil by microalgal lipid	10, 34.9, and 58.1 g/kg	Larval kuruma shrimp (*Marsupenaeus japonicus*)	(1) Microalgal lipid increased survival rate of larval shrimp to 77.0-79.8%; (2) Final body weight of larval shrimp decreased with the substitution of fish liver oil by microalgal lipid.	(4)
*Tetraselmis suecica*	/	2.5, 5.0, and 7.5 g/kg	Pacific white shrimp (*Litopenaeus vannamei*)	(1) Dietary intake of diet with marine microalgae significantly improved survival rate and weight gain while reduced FCR; (2) Addition of 7.5 g/kg marine microalgae in diet down-regulated gene expression of SOD *^*h*^* and GPX *^*i*^*.	(107)

^*a*^ PUFA, Polyunsaturated fatty acid; ^*b*^ SGR, Specific growth rate; ^*c*^ FCR, Feed conversion ratio; ^*d*^ EPA, Eicosapentaenoic acid; ^*e*^ DHA, Docosahexaenoic acid; ^*f*^ DFI, Daily feed intake; ^*g*^ MUFA, Monounsaturated fatty acid; ^*h*^ SOD, Superoxide dismutase; ^*i*^ GPX, Glutathione peroxidase.

In the view of the present authors, to select the most appropriate form of microalgal lipid for aquafeed production, a couple of critical factors, including economic viability, operational efficiency in feed manufacturing processes, and digestive adaptability, should be taken into consideration. It should also be noted that due to the diverse compounds in microalgae, the addition of biomass powder in aquafeed could not only replace fish oil or vegetable oil, but also substitute other nutrients. For example, in the experiment of culturing pacific white shrimp, supplementation of *Nannochloropsis* sp. meal in diet not only reduced the inclusion levels of cod liver oil and soy oil, but also lowered the contents of casein, corn starch, vitamins and so on (106).

#### 4.1.2 Growth performance and health status of aquatic animals

The addition of microalgae-derived lipids in feed exerts complex effects on aquatic animals, but overall improves their growth performance. Many studies reported the positive effects of diet containing marine microalgal lipid on the growth rate and weight gain of aquatic animals, such as gilthead sea bream, Pacific white shrimp, and European sea bass (105, 107, 108). As shown in Table 4, not only the extracted lipid, but also biomass powder in aquafeed has been proven to be favorable to the growth of aquatic animals. In the application of microalgae-based aquafeed, inclusion level is an important factor that can determine the specific effects on growth performance of aquatic animals. In the study of culturing Atlantic salmon, compared with the control group, high inclusion levels (100 and 200 g/kg) of marine microalga, *Pavlova* sp., resulted in lower weight gain (109). Another negative case is that supplementing *Nannochloropsis* sp. powder in aquafeed resulted in diminished growth performance of postlarvae kuruma shrimps (4). When the inclusion level of biomass powder was 10 and 70 g/kg, weight gain and SGR of shrimp were reduced to 188.2%−226.0% and 2.8%−3.2%/day, respectively (4). In the view of the present authors, adverse effects of biomass powder on the growth of aquatic animals are mainly attributed to the degradation-resistant cell wall and anti-nutritional factors in microalgae. Particularly, this weakness becomes amplified during the aquaculture of carnivorous fish species, which could not efficiently digest cellulose-rich cell wall of microalgae. Therefore, to apply microalgal lipid as an alternative of fish oil or vegetable oil for aquafeed production, pretreatment must be performed to attenuate potential adverse effects of microalgal compounds on aquatic animals.

Positive effects of microalgal lipid or biomass powder on the health status of aquatic animals mainly include the enhancement of immunity systems and improvement of biochemical properties. Firstly, immunity systems of aquatic animals can be adjusted by the dietary intake of microalgal lipid or biomass powder, thus improving the stress resistance and survivability of aquatic in adverse environmental conditions. For example, white blood cells, lymphocyte, granulocyte, and red blood cells of *Trachinotus ovatus* fed marine microalgae-supplemented diet were improved (21). Besides, the levels of SOD, CPX, and some other antioxidant enzymes in aquatic animals can be impacted by the dietary intake of marine microalgae (107). Secondly, some biochemical parameters, which determine the health status of aquatic animals, are influenced by marine microalgae-supplemented diet. The improvement of PUFA content in fish tissue has been widely documented (Table 4). Also, significant drop of triglyceride concentration in the plasma of olive flounder was reported when soybean oil in diet was replaced by the biomass powder of *Schizochytrium* sp. and *Nannochloropsis* sp. (110). In aquaculture practice, the improvement and of biochemical parameters not only benefit the health of aquatic animals, but also enhance the nutritional value of aquatic products. Normally, fish/shrimp products with high level of PUFA are more popular among consumers (111).

### 4.2 Use of microalgae lipid for livestock and poultry farming

#### 4.2.1 Inclusion of marine microalgae in animal feed

Compared with traditional lipid sources, particularly terrestrial plant oil, marine microalgal lipids contain much higher contents of long-chain PUFA (e.g., EPA, DHA, etc.), of which the bio-accumulation in meat, milk, and egg can improve the nutritional values of food products. It was reported that DHA contents in the total fatty acids of salmon oil and flaxseed oil were 3.7% and 0%, respectively, while DHA content in *Schizochytrium* sp. reached 29.4% (112). In addition to PUFA, a couple of lipid-soluble components with high nutritional values are included in marine microalgal lipids. For example, lipid-soluble components crucial for animal's health and metabolism, such as rosmarinic acid, cinnamic acid, gallic acid, and caffeic acid, have been identified in marine microalga, *Dunaliella salina* (113). For example, as a natural additive in animal feed, rosmarinic acid has shown promising results in promoting growth, productive and reproductive performance and improving anti-oxidant status and immunologic indices of livestock (114). Besides, some antioxidants, such as β-carotene, astaxanthin, zeaxanthin, and lutein, are rich in *Dunaliella salina* lipid (115, 116). Although genetic modification techniques have been applied to induce the synthesis of long-chain PUFA in some terrestrial plant oils, the safety of genetically-modified organism remains unclear. At present, microalgae is still a promising source with high safety level providing long-chain PUFA and other natural antioxidants to livestock and poultry.

Marine microalgae with high lipid contents were applied in a variety of forms to provide nutrition to livestock and poultry. In the experiment of employing *Nannochloropsis oceanica* to feed lamb, performances of spray-dried biomass, freeze-dried biomass, and microalgal oil were assessed according to the EPA protection (117). The results showed that EPA content in the rumen of lamb fed freeze-dried microalgae was about 50% higher than that of lamb fed spray-dried microalgae and microalgal oil, demonstrating freeze-dried biomass as a natural rumen-protected source of EPA to ruminants (117). It should be noted that in some cases, even when the same pretreatment method is applied to different marine microalgal species, it can result in different fatty acid utilization rates. For example, it was observed that EPA disappearance of freeze-dried *Nannochloropsis oceanica* and freeze-dried *Phaeodactylum tricornutum* reached 44% and 83%, respectively, by the end of 24-h *in vitro* batch incubation with strained rumen fluid of fistulated sheep (118). Therefore, to maximize the efficacy of marine microalgae in livestock and poultry farming, microalgal species and their supplementation forms in animal feed requires focused attention.

#### 4.2.2 Growth performance of livestock and poultry

As shown in Table 5, marine microalgae have been widely adopted for livestock and poultry farming, exerting favorable effects on animal's growth performance and health status. Firstly, dietary supplementation of marine microalgal lipid or microalgal biomass powder can improve the growth rate of livestock and poultry and reduce FCR. In the study of adding DHA-rich *Schizochytrium limacinum* in feed for lamb feeding, the increase of total weight gain from 9.2 to 11.9 kg was observed during the 7-week experiment (119). Simultaneous increases were also observed in the following parameters, daily gain, growth rate, and feed efficiency (119). Promoting effects of lipid-rich marine microalgae on animals were also reported in the studies of supplementing *Dunaliella salina* for quail feeding and adding *Schizochytrium* sp. for broiler feeding (112, 113). Secondly, immune response of animals can be enhanced by the addition of marine microalgae in feed. The experiment, which supplemented *Nannochloropsis oculata* in Barki sheep's diet, revealed the up-regulated expression pattern of immune and antioxidant markers in ewes post-lambing and their newly born lambs (120). Thirdly, health status of livestock and poultry fed marine microalgae can be improved through the optimization of blood physicochemical parameters, improvement of PUFA in retinal tissues, and so on (2, 121). In the study of feeding broiler with *Dunaliella salina* and *Spirulina* sp., the contents of total cholesterol, low-density lipoprotein, and malondialdehyde in blood samples were reduced, reflecting the improvement of health status of broiler (2). The increase of EPA content was observed in retinal tissues of lamb fed *Nannochloropsis oceanica* (121).

**Table 5:** Application of marine microalgal lipid as feed ingredient for livestock and poultry farming.

**Microalgae**	**Addition amount**	**Aquatic animal**	**Specific effects**	**Reference**
*Schizochytrium limacinum*	5 g/day	Lamb	(1) Compared with basal diet, microalgae contained much more PUFA *^*a*^* (62.5%), particularly DHA *^*b*^* (38.5%) and arachidonic acid (1.0%). (2) Feed efficiency and growth rate of lamb were improved by the supplementation of marine microalgae; (3) Blood cholesterol content tended to decrease (5.6%) in lambs fed marine microalgae; (4) Dietary supplement of microalgae improved total weight gain from 9.2 kg to 11.9 kg; (5) Dietary supplementation of marine microalgae did not change the concentrations of diamine oxidase, glutathione peroxidase, and free malondialdehyde.	(119)
*Nannochloropsis oceanica*	123 g/kg of spray-dried biomass; 92 g/kg freeze-dried biomass; and 12 g/kg of microalgal oil	Lamb	(1) EPA contents in rumen, abomasum, and cecum of lamb fed freeze-dried marine microalgae were the highest; (2) PUFA contents in rumen and abomasum of lamb fed freeze-dried microalgae and microalgal oil were significantly higher than that of lamb fed spray-dried microalgae; (3) Freeze-dried marine microalgae is a natural rumen-protected source of EPA *^*c*^* to ruminants.	(117)
*Nannochloropsis oceanica*	123 g/kg of spray-dried biomass; 92 g/kg freeze-dried biomass; and 12 g/kg of microalgal oil	Lamb	(1) Dietary supplementation of marine microalgae or microalgal oil improved daily fatty acid intake, particularly PUFA intake, of lamb; (2) In hippocampus and prefrontal cortex, brain fatty acid profile remained unchanged, with little alteration in docosapentaenoic acid enhancement; (3) Retinal tissues were particularly responsive to the dietary intervention, with a 4.5-fold enhancement of EPA in the lambs fed freeze-dried marine microalgae compared with the control lambs.	(121)
*Nannochloropsis oculata*	750 g concentrate feed mixture with 10 g/kg microalgae per head each day	Barki sheep	(1) Dietary supplementation of *Nannochloropsis oculata* significantly up-regulated the expression pattern of immune and antioxidant markers in ewes post-lambing and their newly born lambs; (2) With the supplementation of microalgae, stillbirth of the newly born lambs was reduced from 30% to 10% and lamb birth weight was improved from 2.9 to 3.3 kg; (3) In ewes post-lambing, dietary intake of microalgae improved the contents of white blood cells and red blood cells and the activities of glutathione peroxidase and catalase, while reduced malondialdhyde content.	(120)
*Schizochytrium* sp.	1%, 2%, and 3%	Lamb	(1) Inclusion of marine microalgae in diet reduced the ratios of *n*-6:*n*-3 in the fatty acid profiles of perirenal adipose tissue, skirt muscle, and subcutaneous adipose tissue of lambs; (2) Increase of marine microalgae supplementation in diet significantly improved the concentration of total PUFA in the subcutaneous adipose tissue of growing lambs; (3) Dietary intake of marine microalgae did not increase the concentration of PUFA in the skirt muscle of lambs; (4) Neither wool production nor quality was affected by dietary inclusion of marine microalgae in diet.	(153)
*NA* ^a^	8, 15, and 23 g/kg	Dairy sheep	(1) Supplementation of marine microalgae significantly improved PUFA content while reduced SFA *^*d*^* content in milk; (2) Addition of marine microalgae improved the concentrations of some bio-active components in milk.	(127)
*Schizochytrium* sp.	100 and 200 g/bull/day	Qaidamford cattle	(1) Addition of microalgae in diet significantly increased the total antioxidant capacity in meat; (2) Microalgae supplementation increased the contents of PUFA, EPA, and DHA, while reduced the ratio of *n*-6/*n*-3 fatty acid; (3) Microalgae supplementation in diet did not have significant effects on the color parameters, such as lightness, redness, and yellowness, of physicochemical meat quality; (4) Sensory characteristics, including initial juiciness, sustained juiciness, flavor intensity, initial tenderness, sustained tenderness, of the beef from cattle fed microalgae-supplemented diet were improved.	(125)
*Schizochytrium limacinum*	0.144 kg/cow/day	Dairy cow	(1) From Week 4 to Week 6, milk yield (35.4–38.5 kg/cow/day) of dairy cow fed marine microalgae was higher than that of cow in control group; (2) Dietary supplementation of marine microalgae improved the concentrations of PUFA and MUFA *^*e*^* in cow milk to 6.1 and 36.4 g/100 g FA *^*f*^*, respectively; (3) Cheese made with the milk from cow fed microalgae-supplemented diet had higher concentrations of PUFA (5.5 g/100 g FA) and MUFA (35.3 g/100 g FA); (4) Dietary intake of marine microalgae reduced the ratio of *n*-6:*n*-3 in the fatty acid profile of milk and cheese.	(128)
*NA*	200 g/day (24% DHA of total FA)	Dairy cow	(1) Cow fed diet with DHA-rich microalgae had higher body weight in the experimental period; (2) Mammary gene expression was regulated by the dietary intake of marine microalgae.	(1)
*Schizochytrium* sp.	170 and 255 g/day	Dairy cow	(1) Dietary intake of marine microalgae resulted in lower concentrations of total PUFA, but increased DHA concentration in milk; (2) With the increase of addition amount of marine microalgae, the ratio of *n*-3:*n*-6 in the fatty acid profile of milk; (3) DHA transfer efficiency from feeding microalgae to milk fell in a range of 10.1%−11.3%; (4) Dietary supplementation of marine microalgae did not have significant treatment effect on most of the blood hematological and biochemical parameters, except for platelets and thrombocytosis.	(123)
*NA*	50, 100, and 150 g/day	Dairy cow	(1) Milk yield was improved from 18.0 to 19.3 kg/day with the increase of daily supplementation of algae; (2) Substitution of fish oil with algae had no effect on milk FA composition.	(5)
*Schizochytrium* sp.	1.8 kg/day microalgae concentration (corresponding to 40 g DHA/day)	Dairy cow	(1) Dietary supplementation of marine microalgae created a milk fat depression but could not improve the energy balance.; (2) As measured by thiobarbituric acid reactive substances, feeding of DHA-rich microalgae significantly increased lipid peroxidation.	(124)
*Dunaliella salina*	0.25, 0.50, and 1.00 g/kg	Japanese quail	(1) Compared with the control diet, diet with 0.5 and 1.0 g/kg microalgae significantly improved live body weight and body weight gain; (2) FCR was significantly reduced by the dietary supplementation of 0.5 and 1.0 g/kg microalgae; (3) Dietary supplementation of microalgae reduced the contents of total cholesterol and triglycerides; (4) With the addition of microalgae in diet, high density lipoprotein content increased while low density lipoprotein decreased.	(113)
*Schizochytrium* sp.	20 g/kg	Broiler	(1) Broiler fed diet with marine microalgae had the highest body weight and feed intake values; (2) Dietary supplementation of marine microalgae had no effect on FCR *^*g*^*; (3) Meat of broiler fed diet with marine microalgae had the highest content of DHA; (4) Compared with salmon oil and flaxseed oil, marine microalgae resulted in a lower content of thiobarbituric acid reactive substances in the thigh meat in broiler chicken.	(112)
*Dunaliella salina* and *Spirulina* sp.	0.5, 1.0, 1.5, and 2.0 g/kg (1 *Dunaliella salina*:1 *Spirulina*)	Broiler	(1) The lipid profile of broiler was improved through a reduction of total cholesterol and low-density lipoprotein contents; (2) Malondialdehyde contents in the blood samples of broilers fed 0.5, 1.0, and 1.5 g/kg microalgae were lower than that in control group; (3) Dietary supplementation of microalgae reduced the contents of *Escherichia coli* and *Salmonella*, but increase the content of lactic acid bacteria in the cecal fresh content of broiler; (4) Activities of digestive enzymes, including amylase, lipase, and protease, in broilers fed 1.0 g/kg microalgae were significantly higher than the activities of digestive enzymes of broilers in the control group.	(2)
*Schizochytrium limacinum*	0.25, 0.50, 0.75, and 1.00%	Laying hen	(1) Microalgae supplementation in diet significantly improved the content of PUFA in eggs and reduced the ratio of *n*-6/*n*-3; (2) Microalgae did not have significant effects on average daily feed intake, FCR, egg weight, and shell thickness.	(122)
*Schizochytrium* sp.	1.6% and 3.2% microalgae powder; 0.8% microalgae power plus 0.3% microalgae oil	Laying quail	(1) The 0.8% microalgae powder plus 0.3% microalgae oil group exhibited a reduction in daily egg-laying rate and egg mass, alongside an increased FCR; (2) Compared with the control group, supplementation of 3.2% microalgae powder significantly decreased the contents of total cholesterol and triacylglycerol in serum lipids of quails; (3) Dietary intake of microalgae powder and microalgae oil significantly improved the contents of PUFA, DHA, and *n*-3 PUFA in quail egg yolks.	(130)
*Dunaliella salina* and *Spirulina platensis*	0.5, 1.0, 1.5, and 2.0 g/kg	Laying Japanese quail	(1) Addition of microalgae had no significant effect on egg production, egg weight, and egg mass; (2) Microalgae supplementation in diet significantly improved body weight and feed intake of laying quails but had no significant effect on FCR; (3) In terms of fertility and hatchability, 1.0 g/kg is the optimal addition amount of microalgae in quail diet; (4) Dietary intake of microalgae reduced the contents of total cholesterol and triglycerides while did not affect IgG and IgM levels in laying quails.	(129)
*Dunaliella salina*	0.50, 1.00, and 1.50 g/kg	Laying hen	(1) Dietary intake of microalgae (15 days) reduced FCR to 2.04-2.27 and improved egg production to 77.9-88.6%; (2) The contents of albumin and globulin in eggs were improved by microalgae-supplemented diet; (3) Microalgae supplementation in diet improved carotenoid content in egg yolk and increased DHA content in egg.	(116)
*Dunaliella salina*	0.25, 0.50, 0.75, and 1.00%	Laying hen	(1) In three experimental periods (Week 40–44, Week 44–48, and Week 48–52), egg weight was improved with the increase of microalgae supplementation while FCR was reduced; (2) Dietary intake of marine microalgae improved albumen weight, yolk weight, and yolk index of chicken eggs; (3) Supplementation of marine microalgae in chicken diet significantly increased total carotenoids content and intensity of red of egg yolk while slightly reduced luminosity of yolk; (4) Inclusion of marine microalgae in chicken diet provided a linear increase in intestinal villus height and ratio of crypt depth/villus height in the duodenum segments and ileum.	(115)

^*a*^ PUFA, Polyunsaturated fatty acid; ^*b*^ DHA, Docosahexaenoic acid; ^*c*^ EPA, Eicosapentaenoic acid; ^*d*^ SFA, Saturated fatty acid; ^*e*^ MUFA, Monounsaturated fatty acid; ^*f*^ FA, Fatty acid; ^*g*^ FCR, Feed conversion ratio.

Although dietary intake of marine microalgae or microalgal lipid could improve growth performance and health status of livestock and poultry, some exceptional cases should not be neglected. For example, in the study of adding DHA-rich *Schizochytrium limacinum* for laying hen feeding, significant effects of microalgae supplementation on FCR and daily feed intake were not observed (122). Additionally, in the experiment of using *Schizochytrium* sp., for cow feeding, significant treatment effect on most of the blood hematological and biochemical parameters were not reported (123). In practical applications, if marine microalgae or microalgal lipids are added without producing any significant effects on animal's growth and health, this would increase animal farming costs and complicate feed production processes.

It should also be noted that dietary intake of marine microalgae even have negative effects on the growth or health status of animals sometimes. For example, it was reported that lipid peroxidation in the plasma of cow fed PUFA-rich *Schizochytrium* sp. was more serious than that of cow fed basal diet. This phenomenon is probably attributed to the high content of PUFA with the dietary intake of marine microalgae (124). Therefore, the comprehensive impacts of incorporating marine microalgae or microalgal lipids into animal feed should undergo thorough evaluation.

#### 4.2.3 Quality of meat, dairy products and poultry eggs

Due to the enrichment of unsaturated fatty acids and lipid-soluble components in marine microalgal lipids, dietary intake of marine microalgae can change the physicochemical and sensory properties of meat. Previous study reported the improvement of initial juiciness, sustained juiciness, flavor intensity, initial tenderness, and sustained tenderness of the beef from cattle fed *Schizochytrium*-supplemented diet (125). In addition, when the supplementation of marine microalgae in animal feed increased from 0 to 200 g/bull/day, the contents of PUFA, including DHA, EPA, and α-Linolenic acid (ALA), in fresh meat were significantly improved (125). Carotenoids from microalgae added into feed could also accumulate in the meat of animals (126). Compared with conventional meat products, those enriched with high-value components or possessing superior sensory characteristics demonstrate significantly higher consumer preference in the market.

PUFA and lipid-soluble components not only exert influence on meat quality, but also transfer from marine microalgae to milk through animal's metabolisms. Compared with the milk (5.5 g/100 g fatty acid methyl esters and 4.1) from ewes fed basal diet, that from ewes fed diet with 23 g/kg marine microalgae contained significantly higher PUFA content (8.9 g/100 g fatty acid methyl esters and *n*-6:*n*-3 ratio of 4.2) and lower ratio of *n*-6:*n*-3 fatty acid (2.1) (127). In the processing of using *Schizochytrium*-based diet to feed dairy cows, 10.1%−11.3% DHA in microalgae was transferred into the milk (123). In addition to milk, dairy products made with milk can be impacted by the animal feed supplemented with marine microalgae or microalgal lipids. It was reported that PUFA content and ratio of *n*-6:*n*-3 fatty acid in the cheese made with milk from diary cow fed DHA-rich *Schizochytrium limacinum* were 5.5 g/100g of total fatty acid and 6.9, respectively (128). By contrast, in the the cheese made with milk in control group, PUFA content was only 3.9 g/100g of total fatty acid and ratio of *n*-6:*n*-3 fatty acid reached as high as 8.1 (128).

In the studies of supplementing marine microalgae for poultry farming, the improvements of laying rate and poultry eggs' quality were widely reported (Table 5). Fertility and hatchability of laying quails fed diet with 1 g/kg microalgae reached 96.7% and 73.3%, respectively, which were much higher than those in control group (129). In addition, dietary supplementation of *Dunaliella salina* increased albumen weight, yolk weight, and yolk index of chicken eggs (115). Intensity of red of egg yolk was also improved, showing the influence of natural pigments in marine microalgae on the egg quality (115). The increases of carotenoid content and DHA content were observed in the eggs of laying hens fed *Dunaliella salina*-supplemented diet (116). Enrichment of PUFA in egg yolks was also observed in another study which supplemented *Schizochytrium* sp. and its lipid in diet for laying quails feeding (130).

Dietary intake of dairy products and eggs with high nutritional values mentioned above will finally impact the health of consumers. For example, in the context of carotenoids' unique antioxidative properties, dietary carotenoid intake has beneficial effect on the treatment of some widespread modern civilization diseases, such as cardiovascular, cancer, or photosensitivity disorders (131). In addition, the importance of long-chain PUFA intake has been intensively reported in patients with major depressive and bipolar disorders, amyotrophic lateral sclerosis, Parkinson's disease, and Alzheimer's disease (132). Therefore, supplementation of microalgal lipid in animal feed can indirectly exert a positive impact on consumer health by increasing the antioxidant content in dairy, meat, and egg products.

## 5 Challenges and prospects

### 5.1 Challenges

Groundbreaking advancements in three pivotal domains, fundamental research on marine microalgal lipid metabolism, applied research on lipid-rich marine microalgae cultivation, and industrialization research on lipid utilization in aquaculture and livestock/poultry farming, have paved the way for the comprehensive utilization of marine microalgal lipid. However, there are still some challenges that merit the attentions of researchers and policy-makers. Due to the existing challenges, substitution of fish oil or vegetable oil by microalgal lipid as an ingredient of animal feed remains far from industrial-scale application. Therefore, researchers should abandon overly optimistic attitudes and address existing technical challenges, further advancing the application of microalgal lipids in the feed industry through innovation.

#### 5.1.1 Safety risks of marine microalgal lipid from wastewater

As discussed above, wastewater, particularly food processing wastewater and agricultural wastewater, can be exploited as an alternative of artificial culture medium to cultivate marine microalgae for lipid production. However, potential safety risks of marine microalgal lipid produced in wastewater limit the use of lipid in downstream industry. Firstly, due to the presence of various negatively charged functional groups on microalgal cell surfaces, marine microalgae exhibit a strong capacity for metal ion adsorption and enrichment (133, 134). For example, in the culture medium with 4 and 8 mg/dm^3^ chromium and 5 and 10 mg/dm^3^ plumbum, three marine microalgae, *Amphora coffaeiformis, Navicula salinicola*, and *Dunaliella salina*, isolated from Tunisian coasts absorbed around 90% chromium and *Dunaliella salina* absorbed over 90% plumbum (135). Although food processing wastewater and agricultural wastewater might contain trace amounts of heavy metals, their bio-accumulation by marine microalgae can still adversely affect the safety of microalgal lipid products, ultimately compromising consumer health through the food chain (136, 137). Secondly, rapid proliferation of airborne pathogenic microorganisms in the nutrient-rich wastewater during marine microalgae cultivation is also one of the primary limiting factors constraining the utilization of wastewater as a culture medium alternative. Therefore, enhancing the safety of marine microalgae biomass harvested from wastewater will play a pivotal role in enabling the widespread application of this novel model.

#### 5.1.2 Poor digestibility and palatability of marine microalgae

The cell walls of most marine microalgae contain various components that are difficult to digest, which results in poor digestibility and absorption of marine microalgae products by carnivorous fish. In aquaculture practice, to attenuate the negative effects of microalgae-supplemented diets on aquatic animals' digestive metabolism, inclusion ratios of marine microalgae biomass in aquafeed were controlled at a low level. In some publications, inclusion ratios of marine microalgae biomass in aquafeed for *Trachinotus ovatus, Paralichthys olivaceus*, and *Marsupenaeus japonicus* were lower than 10% (4, 21, 110). By contract, excessive addition of marine microalgae biomass in aquafeed not only negatively affects the digestion of aquatic animals, but also results in the nutrient wastage in marine microalgae.

Some marine microalgae have off-flavors that compromise feed palatability, thereby diminishing the feed intake of animals, particularly the animals which do not feed on marine microalgae in the natural environment. It was discovered that the excessive supplementation of microalgae biomass in aquafeed resulted in the decrease of feed intake of *Micropterus salmoides* (138). In addition to the natural off-flavors, deterioration of marine microalgae during the transportation, processing, and storage could also result in the poor palatability of microalgae-supplemented feed. For example, in the process aquafeed extrusion, high temperature and pressure may accelerate the peroxidation of PUFA in microalgal lipid and intensify the rancid odor of lipid (139).

#### 5.1.3 Uncertainty of applying new models for marine microalgae cultivation

Rotating algal biofilm and filamentous fungal pelletization mentioned above have been widely adopted to immobilize freshwater microalgae, however, their applications in the immobilization and cultivation of marine microalgae remains highly uncertain.

Performance of marine microalgae and bacteria/fungi in EPS secretion, which determines the formation of biofilm, under high-salinity conditions has not been fully studied. As an extreme environmental condition, high salinity may negatively impact microbial survival and metabolism, thereby suppressing the formation of microalgal biofilms. In addition, high salinity exhibits significant corrosiveness, imposing stringent requirements on mechanical strength and corrosion resistance of the attachment substratum. Consequently, traditional rotating algal biofilm employed for freshwater microalgae immobilization may require structural and material modifications to adapt them for the immobilized cultivation of marine microalgae. Up to now, few studies have evaluated the performance of rotating algal biofilm in the immobilized cultivation of marine microalgae.

Most filamentous fungi were employed to immobilize freshwater microalgae through pelletization. Only few studies have evaluate the feasibility of using fungal pellets for marine microalgae immobilization. For example, co-agitation of *Aspergillus oryzae* with marine *Tetraselmis subcordiformis* resulted in the immobilization of 99.5% marine microalgae through adsorption onto mycelial surface (100). However, performance of immobilized marine microalgae on fungal pellets in biomass production and lipid accumulation has been rarely studied. Compared with freshwater microalgae, marine microalgae have totally different culturing conditions. High salinity may negatively impact the metabolisms of filamentous fungi or the surface charge of fungal pellets. Therefore, effects of environmental conditions (e.g., high salinity, low temperature, etc.) that promote marine microalgae growth and lipid accumulation on the metabolic activities of filamentous fungi and the adsorption characteristics of fungal pellets require further investigation.

### 5.2 Prospects

Based on the discussion of this review, marine microalgae lipid holds significant application potential in fields such as carbon capture, feed nutrition, and animal health from the perspective of industrial chain development. Firstly, this review highlights the application prospects of marine microalgal lipid synthesis in the field of carbon emission reduction at an industrial scale, demonstrating its significant importance for achieving carbon neutrality goals. This aspect has often been overlooked. Secondly, this review comprehensively discusses the production conditions of marine microalgal lipids and their applications in the animal feed industry, providing readers with a more intuitive understanding from an industrial chain perspective. In contrast, previous papers have typically focused on only one aspect, seldom analyzing and discussing the topic from a full industrial chain perspective. Thirdly, this review explores the impact of marine microalgal lipid on animal health, which holds significance for achieving sustainable development in aquaculture and livestock farming industries. In summary, by examining the production and utilization of marine microalgal lipid from a full industrial chain perspective, this review presents the tremendous potential of the marine microalgae-based lipid industry to readers.

Some suggestions to solve the aforementioned challenges, promoting the industrial application of marine microalgal lipid in aquaculture and livestock/poultry farming are listed as follows. Firstly, government, which recognizes the dual economic and ecological benefits of using wastewater for marine microalgae cultivation and microalgal lipid production, should develop comprehensive regulatory frameworks governing wastewater selection, pretreatment protocols, and lipid quality control. For example, effluents with high contents of contaminants, such as heavy metals and antibiotic residues, should not be permitted for marine microalgae cultivation, thereby ensuring bio-safety compliance and sustainable lipid production. Secondly, fermentation or enzymatic hydrolysis technology can be adopted to improve digestibility and palatability of marine microalgae-supplemented feeds. Specific enzymes, such as chitinase, lysozyme, pectinase, and sulfatase, have been employed to digest microalgae biomass and degrade algal cell wall (140). In the industrial application, as the cell walls of marine microalgae break down or are disrupted, the components within the cells become more easily absorbed and utilized by animals. It should be noted that the cost of fermentation or enzymatic hydrolysis should be controlled by technological innovation. Otherwise, market acceptance of fermented or hydrolyzed marine microalgae product is low. Thirdly, intensive studies should be conducted to evaluate the survival of filamentous fungi in in marine microalgal cultivation conditions and analyze the relation between filamentous fungi and immobilized marine microalgae in lipid synthesis. Besides, measures can be taken to screen filamentous fungi with high resilience to high-salinity and low-temperature conditions. For example, marine filamentous fungi obtained from polar marine environment can be collected to support the industrial-scale immobilization of marine microalgae for lipid production.

## 6 Conclusion

Marine microalgae have emerged as a transformative solution for sustainable lipid production, offering a viable alternative to traditional lipid sources, such as soybean, peanut, and reduction fishery. This review underscores the unique advantages of marine microalgae-based lipid production in carbon sequestration toward the goal of carbon neutrality. Biosynthesis of lipid, PUFA, and lipid-soluble components in marine microalgae is intricately regulated by environmental factors, such as nutrient availability, temperature, light conditions, and so on, which can be strategically manipulated to improve the nutritional values of microalgal lipids. Technological advancements, including two-stage cultivation models, wastewater-based microalgae cultivation, and immobilization techniques, demonstrate significant potential to reduce production costs and improve scalability of marine microalgae-based lipid production.

The practical applications of marine microalgal lipids in aquaculture and livestock/poultry farming highlight their role in improving growth performance, immune response, and nutritional value of animal-derived products. However, challenges persist, particularly regarding potential safety risks of microalgal biomass, low digestibility due to resilient cell walls, and uncertainty of applying new technologies for marine microalgae production. Addressing these issues requires robust regulatory frameworks for wastewater selection, technological advancements in enzymatic or fermentation-based pretreatments of biomass, and further research into marine-specific immobilization technologies.

Looking ahead, marine microalgae-based lipid production hold immense promise in contributing to global food security, carbon sequestration, and the transition toward a bio-based circular economy. By overcoming existing technical and socioeconomic barriers, marine microalgae can play a pivotal role in achieving the goals of sustainable development, fostering a resilient agricultural system which harmonizes environmental protection with economic development.
